# Cat Scratch Colon: A Case Report and Review of the Literature

**DOI:** 10.7759/cureus.79154

**Published:** 2025-02-17

**Authors:** Tareq Alsaleh, Swotantra Gautam, Nouman Shafique, Prachi Mann, Eduardo Lorenzo, Baha Aldeen Bani Fawwaz, Abu Hurairah

**Affiliations:** 1 Internal Medicine, AdventHealth Orlando, Orlando, USA; 2 Gastroenterology and Hepatology, AdventHealth Orlando, Orlando, USA

**Keywords:** adult gastroenterology, clinical gastroenterology, colonoscopy complications, endoscopy, internal medicine related, microscopic colitis, post-colonoscopy injury, colonoscopy

## Abstract

We present a case of a 63-year-old woman with a history of compensated alcoholic cirrhosis and chronic kidney disease (CKD) who presented with a 3-day history of hematochezia. Initial laboratory results showed severe anemia with a hemoglobin level of 4.3 g/dl. She was then prepared for an esophagogastroduodenoscopy (EGD) and colonoscopy (CSP). The EGD showed moderate inflammation in the antrum and duodenal bulb without signs of bleeding. CSP revealed friable mucosa with spontaneous bleeding in the proximal ascending colon and cecum, indicating a cat scratch pattern. Additionally, non-bleeding prolapsed hemorrhoids were identified. The patient reported resolution of bleeding and tolerated diet advancement. We concluded that the bleeding was likely due to hemorrhoids. The patient was educated on conservative management of hemorrhoids and discharged in stable condition. During follow-up, the patient reported intermittent episodes of rectal bleeding. A repeat CSP conducted 6 months later revealed diffuse mild diverticulosis and non-bleeding prolapsed hemorrhoids were also observed. The histopathology of the colonic mucosa was normal. The patient was referred to a colorectal surgeon to discuss other treatment options for her hemorrhoids.

We have also reviewed the literature related to this endoscopic finding and explored the similarities and differences in our case compared to those reported in the literature. Based on a comprehensive literature review and our patient's clinical course, we propose recommendations regarding the clinical significance and management strategies of CSC. Larger scale studies are needed to better define the underlying pathophysiology, predisposing factors, clinical implications, and optimal management of CSC.

## Introduction

Cat scratch colon (CSC), an endoscopic entity, is characterized by distinct, bright red linear marks in the ascending colon or cecum, resembling a cat's scratch. This term was first used to describe this endoscopic finding by McDonnell et al. in 2007. The speculated cause is barotrauma of the right colon from air insufflation into a colon with preexisting altered compliance [1]. Here, we present a case of this finding in a patient with multiple comorbidities thought to contribute to its formation. A literature review was conducted to better understand this finding and its clinical significance.

## Case presentation

Our case is a 63-year-old African American female with a past medical history of alcoholic cirrhosis, chronic pancreatitis, hypertension (HTN), hypothyroidism, chronic obstructive pulmonary disease (COPD), chronic heart failure (CHF), and chronic kidney disease (CKD) who presented at our facility with hematochezia and anemia. She complained of bright red rectal bleeding, variable in amount, occurring with bowel movements for 3 days. She denied a history of melena, recent alcohol use, or abdominal pain. She has a past surgical history of hemorrhoidectomy with hemorrhoidal artery ligation surgery (HALS). She is a previous alcohol drinker and smoker. Her medications include montelukast, aspirin, simvastatin, levothyroxine, gabapentin, folic acid, potassium chloride, calcium citrate, ambisartan, bumetanide, calcium carbonate, midodrine, thiamine, pantoprazole, a fixed-dose combination of umeclidinium and vilanterol, and ursodeoxycholic acid. She did not report a previous history of colonoscopy.

**Figure 1:**
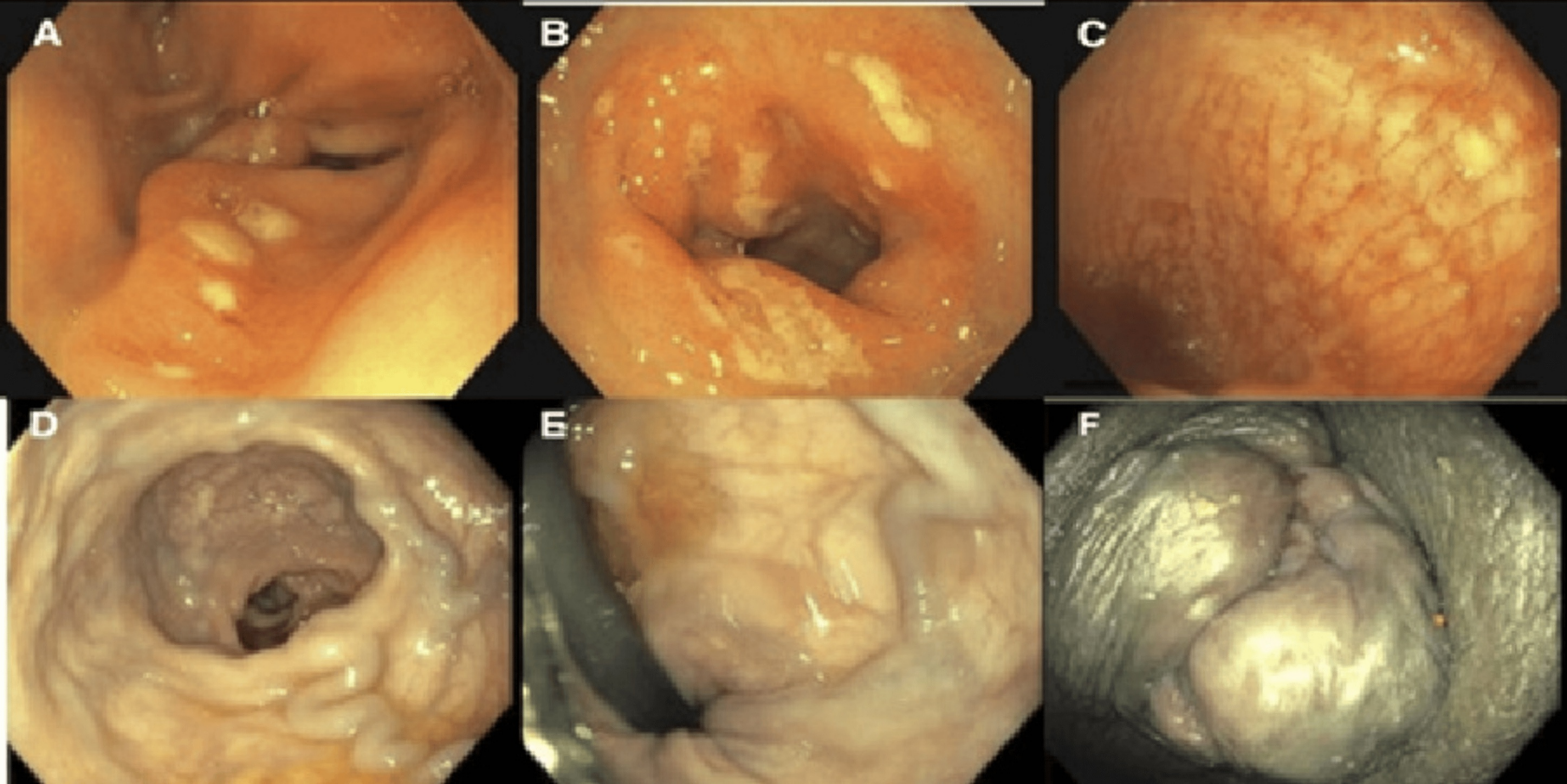
Colonoscopy findings Images (A+B): EGD findings of moderate inflammation in the antrum (A) and the duodenum (B). Image (C): Initial CSP findings of cat scratch lesions in the proximal colon. Images (D+E+F): CSP findings of prolapsed internal and external hemorrhoids.

Her physical examination was unremarkable except for conjunctival pallor and prolapsed external and internal hemorrhoids without evidence of bleeding. Laboratory evaluation showed a hemoglobin level of 4.3 g/dl. Her platelet count was 83,000 cells/mm^3^ with a normal PT/INR (prothrombin time/international normalized ratio). On transthoracic echocardiography (TTE), mild pulmonary hypertension and diastolic dysfunction were seen with a preserved ejection fraction (HFpEF 60-65%). The patient underwent a blood transfusion and was initiated on pantoprazole and octreotide due to initial concerns of variceal bleeding. Endoscopic examination revealed erythematous mucosa in the antrum, with no signs of recent or active upper GI bleed. A colonoscopy revealed friable mucosa with spontaneous bleeding in the proximal ascending colon and cecum, which appeared to resemble a cat scratch pattern (Figure 1). Small polyps were found in the ascending, descending, and sigmoid colon and were resected and retrieved. Mild diverticulosis was present throughout the entirety of the colon except the rectum, along with non-bleeding prolapsed external and internal hemorrhoids.

The colonoscopy was a smooth procedure, and no iatrogenic trauma occurred. The cat scratches were seen as bright erythematous linear marks with spontaneous bleeding in the cecum and ascending colon. The histopathology of the colonic mucosa was normal. The conclusion was that the bleeding was likely due to hemorrhoids. The patient never complained of any gastrointestinal symptoms, such as pain, cramping, diarrhea, or constipation, throughout their hospital stay and prior to presentation. She was educated on dietary and lifestyle modifications for hemorrhoids and prescribed topical lidocaine-hydrocortisone. She was discharged in stable condition. A repeat colonoscopy following complaints of persistent hematochezia after 6 months revealed diffuse mild diverticulosis and non-bleeding prolapsed hemorrhoids with normal histopathology of the colonic mucosa. The patient was then referred to a colorectal surgeon to discuss other treatment options for her hemorrhoids. She continued on non-surgical treatment with a high-fiber diet, stool softeners, sitz baths, and topical hydrocortisone. Her symptoms later resolved after compliance with conservative lifestyle measures.

## Discussion

The prevalence of CSC was found to be 0.19% amongst the 10715 colonoscopies performed in a case series by Baudet et al. A slightly low prevalence has been previously reported by McDonnell et al. where CSC was reported in 21 (0.25%) of the 8277 colonoscopies [1,2]. Nevertheless, this is in opposition to Baudet et al., where a higher prevalence in older males has been depicted [2]. 

The available literature describing CSC was reviewed by searching for the phrase ‘cat scratch colon’ across PubMed, Web of Science, and Ovid. A total of 23 reported cases were found, with varying characteristics summarized in Table 1. The average age of these patients was 67.4 years, and 65.2% of them were females. These findings are consistent with those reported by McDonnell et al., where the mean age was 64, and 81% of patients were female. Our patient, who is an elderly female, also fits this pattern. In that same study, 18/21 (85.7%) biopsies showed normal histology. Among the cases we found, 46.2% (6/13) of the biopsies showed normal histology, which is also similar to our patient [1]. Nevertheless, Baudet et al. reported biopsies that showed diversion colitis in 15/20 (75%) and collagenous colitis in 5/20 (25%) patients. Another difference is that most cases of CSC in that study were males (75%) [2].

**Table 1:** Characteristics of reported cases of cat scratch colon in the literature [3-25]

Characteristic	Percentage of reported	Number reported
Gender	Male 34.8% (Mean age 68.3)	8
	Female 65.2% (Mean age 58.4)	15
Comorbidities	Hypertension 38.9%	7
	Diverticulosis 38.9%	7
	Colorectal Cancer 22.2%	4
	ASCVD 21.4%	3
	Chronic Kidney Disease 21.4%	3
Complications	Pneumoperitoneum 13%	3
Biopsy	Normal 46.2%	6
	Diversion colitis 23.1%	3
	Amyloidosis 7.7%	1
	Colonic spirochetosis 7.7%	1
	Lymphoplasmacytic infiltration 7.7%	1
	Non-specific colitis 7.7%	1
Location of finding	Cecum 33.3%	7
	Ascending 38.1%	8
	Transverse 4.8%	1
	Cecum and ascending 14.3%	3
	Cecum, transverse, and ascending 4.8%	1
	Rectal stump 4.8%	1
Purpose of colonoscopy	Screening 31.8%	7
	Diagnostic 68.2%	15

It is suggested that CSC can represent barotrauma from air insufflation into a less compliant colon during colonoscopy [1]. The biopsy of colonic mucosa from the sites of CSC was normal in our case. This is in accordance with the available literature according to which the etiology of CSC is not well known. Vascular malformations do not seem to play a role, and no signs of inflammation are seen in the biopsies. Further, the lesions are not due to direct scope trauma [1,3]. In contrast, Baudet et al. believe that the mere insufflation of air during colonoscopy should not lead to these lesions in normal mucosa, suggesting that among the patients studied in McDonnell et al, those who developed CSC on normal mucosa might have had conditions that were unnoticed. CSC lesions in Baudet et al. were most commonly associated with diversion colitis, followed by collagenous colitis, a finding consistent with the higher prevalence of collagenous colitis depicted by McDonnell et al [1]. Collagenous colitis is usually macroscopically normal under endoscopic examination, but findings such as ulceration of the vascular mucosal pattern and mucosal defects resembling cat scratches have been reported. In a systematic review involving 1582 patients with microscopic colitis, 7/615 (1.1%) of the patients with macroscopic lesions in the gut had linear streaks similar to CSC [4]. Chronic colitis associated with spirochetosis has also been reported in a biopsy of endoscopic findings of CSC [5]. One of the most common morbidities found in the cases in the literature was diverticulosis, a finding in our patient’s colonoscopy. The association of CSC with a less compliant colon can be linked to diverticulosis which causes airflow limitation and trapping in the colon [6]. 

Another probable correlation of CSC to our case can be drawn from a hypothesis by Purnak et al., according to which an epithelial disruption and tendency to bleed can be due to vitamin A and K deficiency. This explanation is plausible in our case, as our patient has a history of chronic pancreatitis which may lead to a deficiency of fat-soluble vitamins [7]. Our patient is under multiple medications, including aspirin. Although CSC has not been shown to be affiliated with any drug-related events, a hypothesis of intake of nonsteroidal anti-inflammatory drugs as an association with CSC has been postulated by Payeras et al [8]. A significantly increased risk of ischemic colitis was demonstrated among patients with heart failure (HF) in a systematic review and meta-analysis by Wongtrakul et al., which dictates the possibility of direct injury from ischemic colitis due to reduced peripheral blood flow from the low cardiac output in HF. Our patient has a history of diastolic heart failure, suggesting another possible factor for altered compliance of the colonic wall [9].

The most common locations of CSC were the cecum (33.3%) and ascending colon (38.1%), which is consistent with the available literature. Cat scratch colon has been reported in only the cecum and ascending colon until recently where the transverse colon has also been involved [1,2]. One of the reported cases demonstrates these findings in the rectal stump in a patient who underwent colorectal cancer surgery with a colostomy 15 months prior. The rectal stump was erythematous with loss of vascular pattern suggesting diversion colitis, and CSC lesions appeared following air insufflation. Although the location is unusual, the altered elasticity of an inflamed rectal stump and the presence of diversion colitis are possible risk factors for the development of CSC [10]. In another unusual case, collagenous colitis was initially diagnosed from biopsy samples of CSC lesions in a patient with chronic diarrhea. Six months later, the patient presented with refractory symptoms, and a colonic biopsy revealed amyloidosis [11].

There was no clear association between the presence of CSC and colonoscopy-related bleeding events in the cases reported in the literature. Moreover, in the 21 cases reported by McDonnell et al. and the 20 cases reported by Baudet et al., no post-colonoscopy perforations or cases of pneumoperitoneum were reported. From our literature review, pneumoperitoneum was observed in 3 out of the 23 case reports found (13%), which is not unusual considering the postulated process of barotrauma secondary to air insufflation into a less compliant colon in CSC. Although the development of CSC during colonoscopy might be a risk factor for pneumoperitoneum, the procedure can be performed without complications provided it is conducted with the utmost caution [1-3]. However, it is important to remain vigilant for any signs of barotrauma injury in the period following colonoscopy [12]. We believe that the high rates of pneumoperitoneum among individual case reports are affected by publication bias, as it is more likely for individual cases to be written and published when such a complication develops. Further research with large-scale studies is required to better delineate the associated risk and understand the clinical significance of this endoscopic finding.

In one of the cases reported by McDonnell et al., a follow-up colonoscopy conducted 18 months after the initial presentation revealed no evidence of CSC lesions. Similarly, our patient demonstrated resolution of these lesions upon repeat colonoscopy performed 6 months post-initial diagnosis. A notable parallel between these two cases is the absence of histological abnormalities in the biopsy specimens [1]. While these observations suggest a potential lack of association between CSC and chronic colonic disease, it is important to note that this may not be generalizable to cases where CSC lesions are accompanied by underlying histopathological changes.

As demonstrated by cases in the literature in Table 1, more than half of the biopsies of cat scratch marks seen on colonoscopy revealed an underlying pathological process. This highlights the importance of obtaining biopsies when evaluating these patients. 

## Conclusions

Our patient had spontaneous and hemodynamically significant rectum bleeding in the setting of a rare colonoscopy finding of CSC. This case report favors the speculation of cat scratch lesions being more common in females and in cohorts with altered compliance of colon as evidenced by the associated colonic diverticulosis, aspirin use and chronic pancreatitis with alcoholic cirrhosis in our patient. Other factors that could have contributed to her condition include her acute anemia, polypharmacy with intake of NSAIDs, history of smoking, and potential fat-soluble vitamin deficiencies.

CSC should not be disregarded as a benign incidental endoscopic finding. These lesions should be biopsied as they are associated with underlying pathology in many cases. Although the association is still not clear, CSC can also be associated with perforation and pneumoperitoneum. We therefore recommend close monitoring for clinical signs of perforation the day following the colonoscopy. Large-scale studies are needed to better understand this potential association and the underlying pathophysiology, predisposing factors, and appropriate management of this endoscopic sign.
